# Acute osteomyelitis of the patella due to *Pseudomonas aeruginosa* in an immunocompetent child: A case report

**DOI:** 10.1097/MD.0000000000033012

**Published:** 2023-02-17

**Authors:** Yasunari Kamiya, Kenichi Mishima, Tetsuji Tanaka, Kenta Sawamura, Masaki Matsushita, Shiro Imagama

**Affiliations:** a Department of Orthopaedic Surgery, Nagoya University Graduate School of Medicine, Nagoya, Aichi, Japan; b Department of Orthopaedic Surgery, Holy Spirit Hospital, Nagoya, Aichi, Japan.

**Keywords:** patella, pediatric osteomyelitis, *Pseudomonas aeruginosa*, septic arthritis

## Abstract

Plentiful vascularity and lack of the physis are thought to render the patella less vulnerable to osteomyelitis. *Pseudomonas aeruginosa* (PA) is an opportunistic pathogen predominantly affecting immunocompromised hosts. Despite the ubiquitous nature of PA, osteomyelitis of the patella caused by PA has been rarely reported in children.

**Patient concerns:**

A 5-year-old boy who had presented with a prolonged history of the left anterior knee pain following minor trauma was diagnosed with prepatellar bacterial cellulitis and bursitis. Afterward, a focal osteolytic lesion emerged at the ventral surface of the patella despite oral and intravenous antibiotic therapy lasting for weeks. We described clinical presentation as well as medical and surgical management of pediatric patellar osteomyelitis secondary to prepatellar septic bursitis.

**Diagnoses:**

*Pseudomonas aeruginosa*-associated osteomyelitis of the patella. Magnetic resonance imaging of the left knee showed a focal destructive change of the ventral half of the cartilaginous patella and a suprapatellar joint effusion. Bacterial culture from the bursa revealed *Pseudomonas aeruginosa*.

**Interventions:**

Systemic inflammation, patellar osteochondral destruction, and purulent synovial fluid of the knee were prolonged for 6 weeks despite antibiotics use deemed appropriate and reparative surgical debridement, whereas they were eventually resolved with a 6-week course of intravenous ceftazidime and cessation of continuous intracapsular irrigation.

**Outcomes:**

He was clinically asymptomatic at the latest follow-up but exhibited a minor leg length discrepancy <2 cm associated with overgrowth of the affected femur.

**Lessons:**

This is a rare case of *Pseudomonas osteomyelitis* of the patella in a healthy pediatric patient. Uncommon osteochondral sequelae occurred probably because of a protracted arthritis of the affected knee. We would like to emphasize the ineffectiveness of continuous irrigation without antibiotics for *Pseudomonas aeruginosa*-associated osteomyelitis.

## 1. Introduction

Acute osteomyelitis of the patella has rarely been encountered in children, with only 1.1% of incidence of bone involvement reported in a previous literature of case reports.^[1]^ *Pseudomonas aeruginosa* (PA) is a ubiquitous pathogen that can cause opportunistic infections in virtually all tissues.^[2]^ The ability to inhabit and propagate in aqueous environments is the pathognomonic feature of this gram-negative bacillus.^[3]^ The only published case report regarding osteomyelitis of the patella due to PA focused on antibiotics-related complications and lacked radiographic or clinical outcomes.^[4]^ We report a case of a previously healthy 5-year-old boy with acute osteomyelitis of the patella caused by PA that was recalcitrant to multiple debridement and ultimately resolved with change of intravenous antibiotics and cessation of antibiotics-free continuous irrigation. The patient and his parents were informed that data concerning the case would be submitted for publication, and they provided written informed consent.

## 2. Case presentation

**Figure 1.**
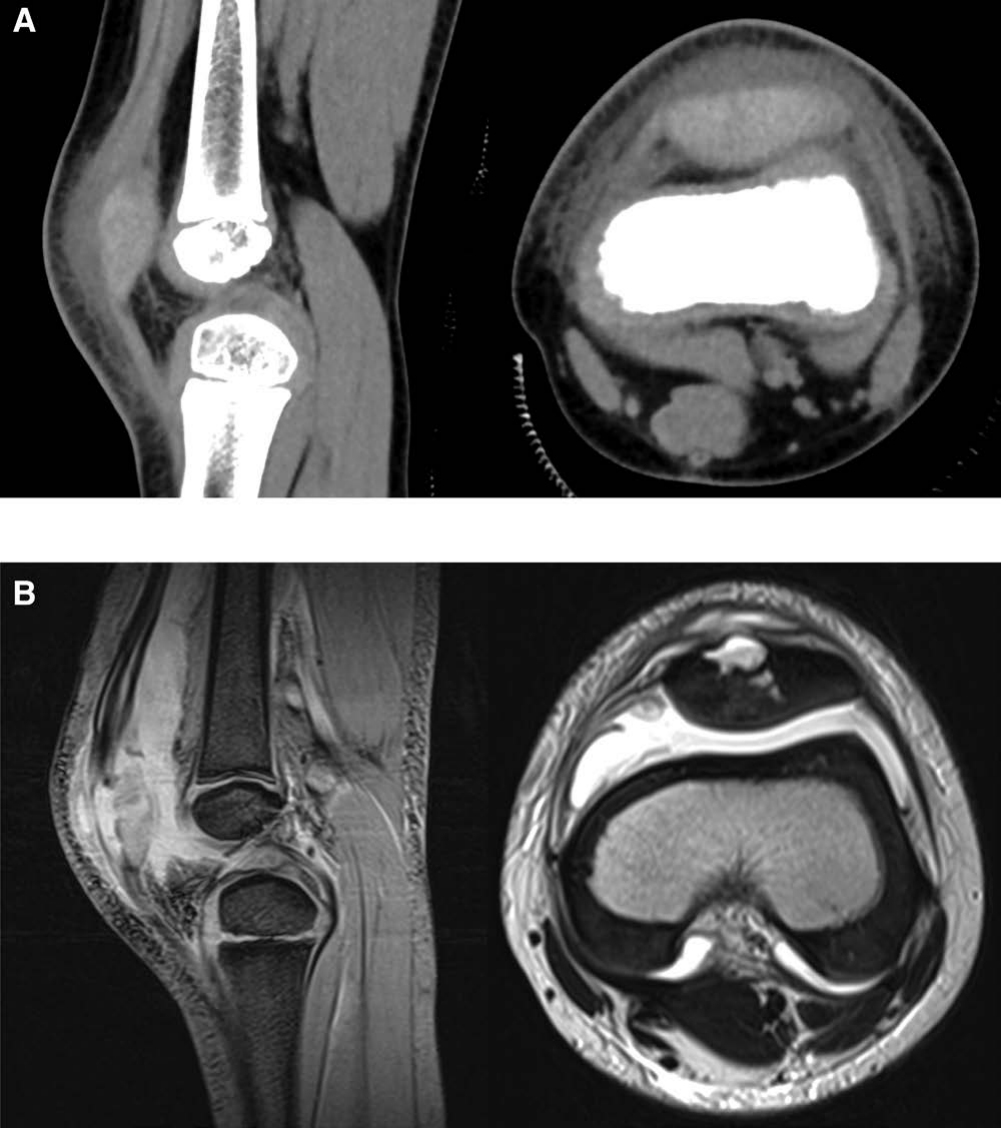
Preoperative computed tomography (CT) and magnetic resonance imaging (MRI) of the left knee. (A) CT sagittal (right) and axial (left) images obtained 6 days after the onset. (B) Short-tau inversion recovery (STIR) sagittal (right) and T2-weighted axial (left) MRI images obtained 21 days after the onset. MRI = magnetic resonance imaging.

**Figure 2.**
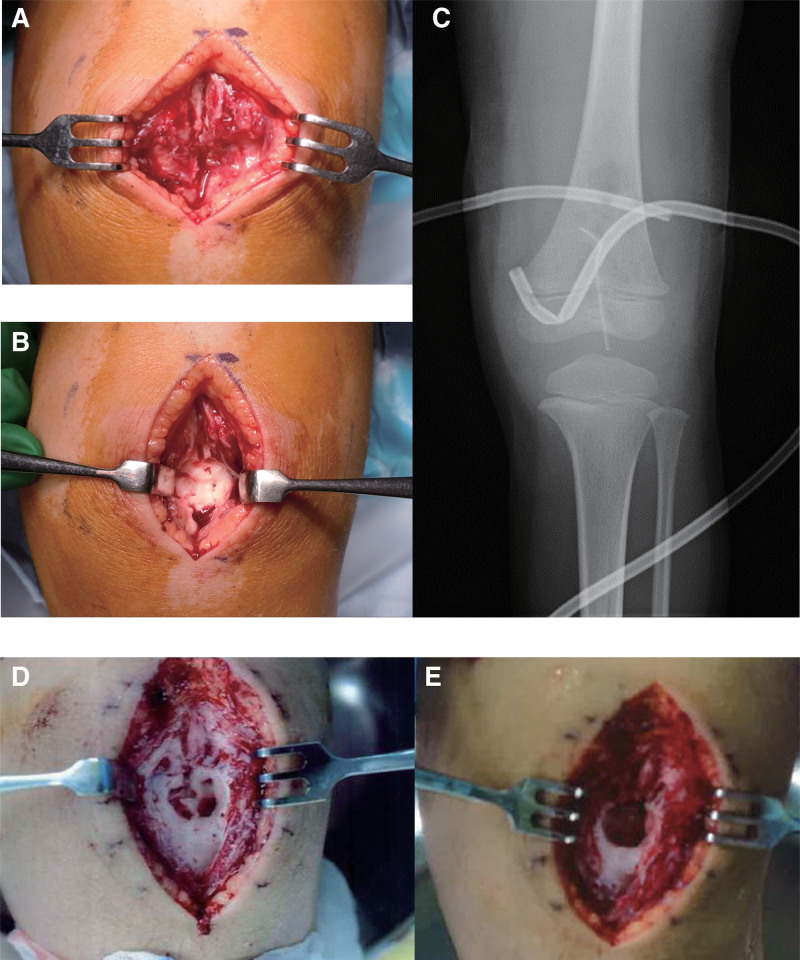
Intraoperative photographs of the left knee. Front view photographs taken before (A) and after (B) curettage of the patellar lesion at the time of the initial debridement. (C) Radiographs made immediately after the initial debridement. Note 2 drainage tubes placed in the suprapatellar pouch for postoperative continuous closed irrigation. Front view photographs taken before (D) and after (E) curettage of the patellar lesion at the time of the second debridement.

**Figure 3.**
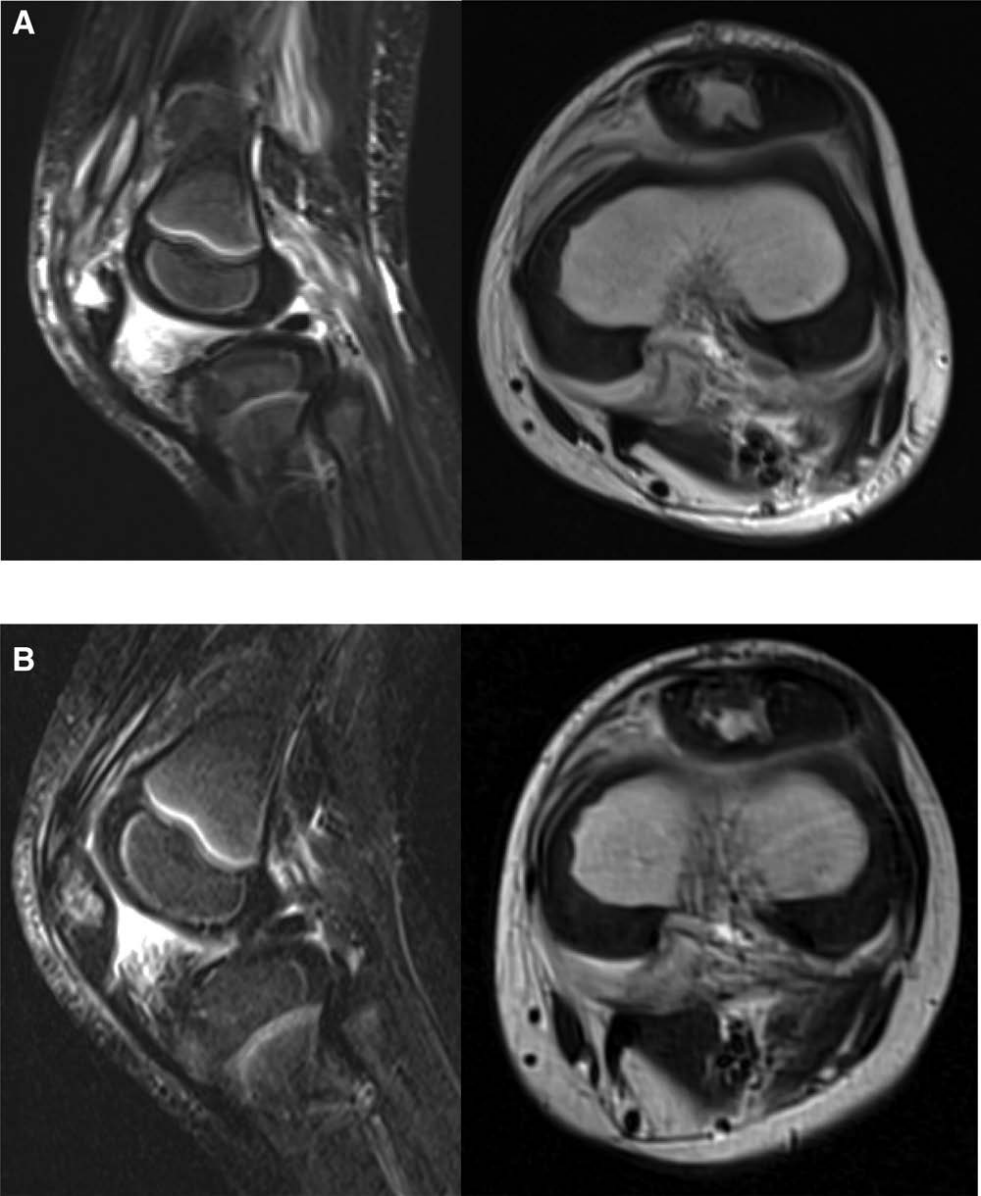
A repeat magnetic resonance imaging (MRI) of the left knee obtained after the referral to our institution. Short-tau inversion recovery (STIR) sagittal (left) and T2-weighted axial (right) MRI images obtained 19 days (A) and 39 days (B) after discontinuation of continuous intracapsular irrigation. MRI = magnetic resonance imaging.

**Figure 4.**
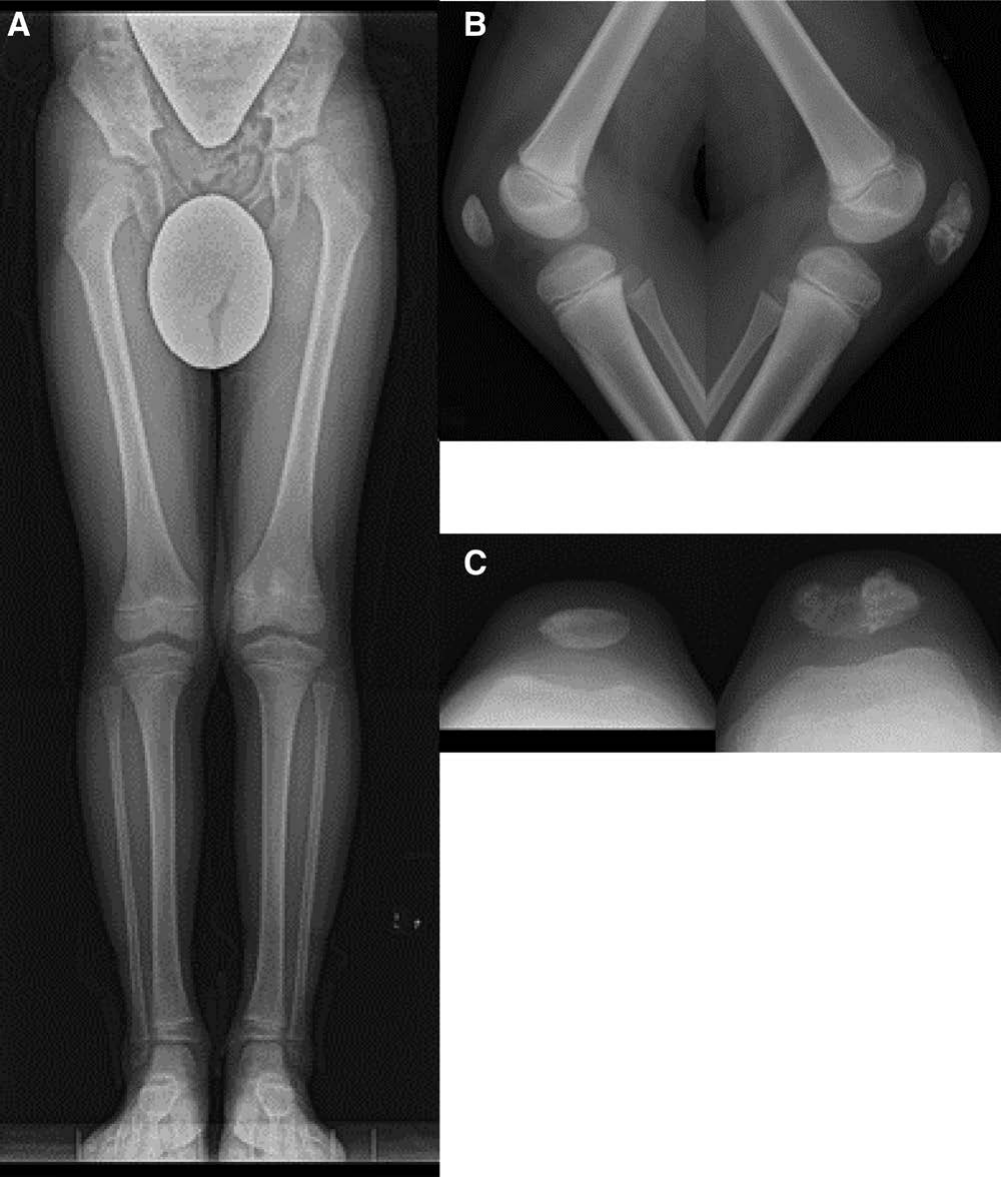
Radiographs obtained 3 years after the onset. (A) A full-length standing anteroposterior radiograph of the lower extremity. Lateral (B) and sky-line (C) views of both knees.

An otherwise healthy 5-year-old kindergarten Japanese boy presented to an emergency department of a community hospital with a 4-day history of the left anterior knee pain, the onset of which occurred while crawling and kneeling in doors. About 1 week earlier, he had sustained a skin abrasion on the anterior aspect of the left knee after a ground level fall. There was localized redness and mild swelling of the anterior knee and dull pain with extreme flexion. Radiographs demonstrated no lytic lesion in the anterior aspect of the patella. He was diagnosed with acute bacterial cellulitis and treated with an orally active third generation cephalosporin. Two day later, he was referred to another community hospital because of persisting symptoms and the occurrence of limping. Computed tomography (CT) showed soft-tissue swelling of the anterior knee, which was interpreted as septic prepatellar bursitis associated with cellulitis, but, in retrospect, exhibited a slight indentation in the ventral aspect of the cartilaginous patella (Fig. 1A). Although oral antibiotic was changed to ampicillin with β-lactamase inhibitor because of suspicion of gram-negative bacterial infections, resting knee pain was further exacerbated. It was not until 2 weeks after the onset of pain that he was started on intravenous antibiotics, with ampicillin and sulbactam administered for 1 day, and then ceftriaxone for 7 days. Magnetic resonance imaging (MRI) showed a focal destructive change of the ventral half of the cartilaginous patella and a suprapatellar joint effusion (Fig. 1B). Blood culture drawn at the time of referral was negative whereas subsequent culture from the bursal fluid aspirate grew PA, suggesting acute purulent Pseudomonas patellar osteomyelitis. He thus underwent urgent debridement of the patella. A longitudinal incision was made over the patella. A subcutaneous abscess was exposed ventral to the patella and thoroughly irrigated. Curettes were used to remove intracartilaginous necrotic tissues of the patella (Fig. 2A and B). The purulent synovial fluid was then evacuated using a vertical incision of the suprapatellar pouch, through which open synovectomy of the patellofemoral joint was performed with forceps and curettes. The incision was irrigated and closed after 2 drainage tubes were placed in the pouch (Fig. 2C). Continuous closed irrigation of the pouch with normal saline was initiated by application of negative pressure suction to one of the tubes. He was transitioned to intravenous meropenem hydrate immediately after the debridement based on culture sensitivities. Cultures taken from the synovial tissues also grew PA. he was made non weight bearing. He continued to have increased white blood cell counts and C-reactive protein values despite surgical debridement with appropriate antibiotic therapy. A follow-up MRI was obtained, which depicted enlargement of the destructive change of the patella and new onset of popliteal effusions (Fig. 2D). He underwent the second debridement the next day. The prior incision was reopened. Gross purulence of the patella was identified and thoroughly cleaned (Fig. 2E). The pouch was irrigated and debrided through the original incision. A second incision was unperformed posteriorly. Postoperatively, continuous closed irrigation of the pouch with normal saline was resumed. Four days later, laboratory evaluation showed an elevated C-reactive protein (CRP) value of 96.9 mg/L and a decreased white blood cell (WBC) count of 2800/μL, and he had a body temperature above 38 °C, suggestive of severe infection. At 6 weeks after the onset, he was transferred to our institution for tertiary care because of uncontrollable infection after debridement twice. On admission, antibiotics therapy was supervised by the infection control team (ICT), and he was started on intravenous ceftazidime empirically. According to the ICT’s recommendations, continuous irrigation was discontinued and the affected limb was immobilized with a splint for comfort. Although a rebound in CRP above 40 mg/L and body temperature above 38 °C was observed 3 weeks after the referral, MRI revealed no progression of cartilaginous destruction of the patella and reduced fluid collections. No bony lesions were seen in the distal femur and the proximal tibia (Fig. 3A). Afterward CRP started to a rapid normalization, descending to <1 mg/L in 1 week, followed by a gradual descent of erythrocyte sedimentation rate (ESR) (Figure S1A and B, Supplemental Digital Content, http://links.lww.com/MD/I510). At 5 weeks after the referral, staged range of motion and weight-bearing exercises of the affected limb was commenced. MRI delineated surrounding edema of the quadriceps tendon and the infrapatellar fat pad (Fig. 3B). He completed a 6-week course of intravenous ceftazidime 1 g 3 times a day. He was not transitioned to oral antibiotics. He was hospitalized for 8 weeks and was discharged walking unaided. No recurrence of swelling, warmth, and tender of the left distal thigh was observed at regular clinical follow-up. Three years after the initial surgery, he still has a slight limitation in both flexion and extension of the left knee. Radiographs demonstrated 1.5 cm of leg length discrepancy owing to overgrowth of the distal end of the affected femur and aberrant ossification of the patella (Fig. 4).

## 3. Discussion

Osteomyelitis of the patella is uncommon condition even in children, with only 1.1% (1/93) of incidence of bone involvement reported in a previous literature of case reports.^[1]^ Pediatric osteomyelitis has been considered to result from hematogenous spread and deposition of bacteria in the metaphysis.^[5,6]^ From an anatomic perspective, the unique microvascular architecture adjacent to the physis, where nutrient capillaries form a loop structure and connects to sinusoids, is thought to contribute to stagnation of blood flow and subsequent bacterial fixation to the sinusoids in growing children.^[7,8]^ The circulation of the patella, which begins at the age of 4 years and maximize at the age of twelve years, is mainly governed by 2 nutrient arteries, 1 penetrating the middle third of the anterior surface and the other entering the lower pole of the bone behind the patellar ligament.^[9,10]^ A multilayer intraosseous anastomosis is composed of surrounding branches of the superior and inferior genicular arteries.^[11]^ These abundant blood supply and the absence of the epiphyseal growth plate seems to make the patella less susceptible to osteomyelitis.^[10]^ Meanwhile, the anterior part of the knee is vulnerable to minor trauma because of the bony prominence of the patella. Although rare in immunocompetent children, frequent abrasion or blunt contusion allows for gradual spread of superficial infection to the most ventral aspect of the patella through the thin overlying soft tissues such as prepatellar bursa, as was seen in this case and others.^[10,12–14]^ Treating clinicians should keep in mind the possibility of the patellar osteomyelitis when local and systemic signs of seemingly cellulitis or bursitis of the anterior knee are prolonged even when it is treated with antibiotics deemed appropriate.

Across all age groups, Staphylococcus aureus has been the most common causative organism, followed by Streptococcus species in pediatric osteomyelitis.^[15,16]^ PA has occupied the third position, accounting for 11% or 10.8% of prevalence.^[17,18]^ A high index suspicion for PA infection is required especially in immunocompromised patients or in cases with preceding trauma involving a puncture wound.^[19,20]^ PA is a ubiquitous organism residing in natural and human environments.^[2]^ Several components of bacterial pathogenicity have been considered to involve the chronicity of infections by PA, such as biofilms, antibiotics resistance, and toxigenic virulences.^[21–23]^ It is noteworthy that PA is capable of long-term survival in water without nutrients by switching to a dormant state.^[3]^ Furthermore, dormancy phenotype in PA have shown to increase antibiotics resistance, biofilm formation, and resistance to chemicals.^[24–26]^ Dormant cells in biofilm populations possess the hallmark of persister cells that are tolerant to high concentrations of antibiotics.^[27]^ In fact, some persister genes have been identified in dormant PAs.^[28]^ We thus suppose that, in the present case, prolonged implementation of antibiotics-free continuous intracapsular irrigation could increase such bacterial pathogenicity of PA as well as attenuate intracapsular concentration of antibiotics that eluted into the knee joint, resulting in failure to manage infection despite surgical debridement and appropriate antibiotic therapy. Since the PA strains isolated from the intraoperative specimens were sensitive to both meropenem and ceftazidime in this case, whether switching from meropenem to ceftazidime, simultaneously performed with drainage removal, had a crucial impact on eradicating the infection is uncertain. According to a report from Kazakhstan that investigated antibiotic susceptibility of PA causing osteomyelitis, its sensitivity to various antibiotics used as empirical therapy, including meropenem and ceftazidime, decreased significantly over a recent 3-year study period.^[18]^ Given the emergence of carbapenem or multidrug-resistant strains and a highly environmental adaptability in PA,^[22,29]^ repeated microbiological test and antibiotic stewardship are requisite for an appropriate choice or switch of antibiotics and successful treatment of this pathology.

Pediatric osteoarticular infection is accompanied by several potential complications from negligible bone growth disturbance and joint contractures up to devastating osteochondral necrosis and joint dislocations.^[30]^ There are several risk factors associated with conspicuous sequelae of this condition, such as diagnostic and therapeutic delay, inadequate antibiotic treatment, and newborn patients. Under such circumstances, involvement of the epiphyseal plate of a long tubular bone can cause partial or total growth arrest, manifesting leg length discrepancy and an axial deviation of the affected limb.^[5]^ In cases of intra-articular spread of the infection, osteochondral necrosis can develop, resulting in refractory joint stiffness or dislocation.^[31]^ Fortunately, thanks to relatively rapid debridement of infectious synovitis, only minor complications, namely, a defect in ossification of the patella and an inability to squat, have developed after clearance of the infection in this case. There has been no sign of premature epiphyseal closure whereas overgrowth phenomenon of the affected femur has been observed so far. To our knowledge, no incidence of apparent leg length discrepancy owing to an overgrowth of the affected side has been reported in septic arthritis of the knee in children. Interestingly, it has been noted that all the patients with juvenile idiopathic arthritis in whom the disease occurred before the age of 9 had overgrowth of the affected limb that had developed within the first 3 or 4 years and never exceeded 3 cm.^[32]^ Prolonged intracapsular inflammation that had been manifested by elevated inflammatory markers and diffuse joint swelling for weeks to months seems to have caused stimulation of the epiphyseal growth plate and hypertrophy of the epiphyseal cartilage around the knee joint in the present case. Continuous follow-up is necessary to determine whether the discrepancy will extend up to the degree requiring epiphysiodesis.

## 4. Conclusion

This is a rare case of *Pseudomonas osteomyelitis* of the patella in a healthy pediatric patient. In patients with osteomyelitis caused by PA, continuous irrigation without antibiotics should be avoided, especially if there is a recurrence of infection despite surgical debridement with appropriate antibiotic therapy.

## Author contributions

**Conceptualization:** Yasunari Kamiya, Kenichi Mishima.

**Data curation:** Tetsuji Tanaka, Kenta Sawamura.

**Supervision:** Masaki Matsushita, Shiro Imagama.

**Writing – original draft:** Yasunari Kamiya.

**Writing – review & editing:** Kenichi Mishima.

## Supplementary Material
