# Supplementary material for: Acute osteomyelitis of the patella due to Pseudomonas aeruginosa in an immunocompetent child: A case report
Source: Medicine (Baltimore). 2023 Feb 17;102(7):e33012. doi: 10.1097/MD.0000000000033012 (PMC9936032; doi:10.1097/MD.0000000000033012)

Supplementary Figure 1. Temporal profile of inflammatory markers and overview of antibiotic treatment. Time series data before (A) and after (B) the referral to our institution. CRP, C-reactive protein; ESR, erythrocyte sedimentation rate; WBC, white blood cell counts; CDTR-PI, cefditoren pivoxil; CVA/AMPC, clavulanic acid/amoxicillin; ABPC/SBT, ampicillin/sulbactam; CTRX, ceftriaxone; MEPM, meropenem; CAZ, ceftazidime.

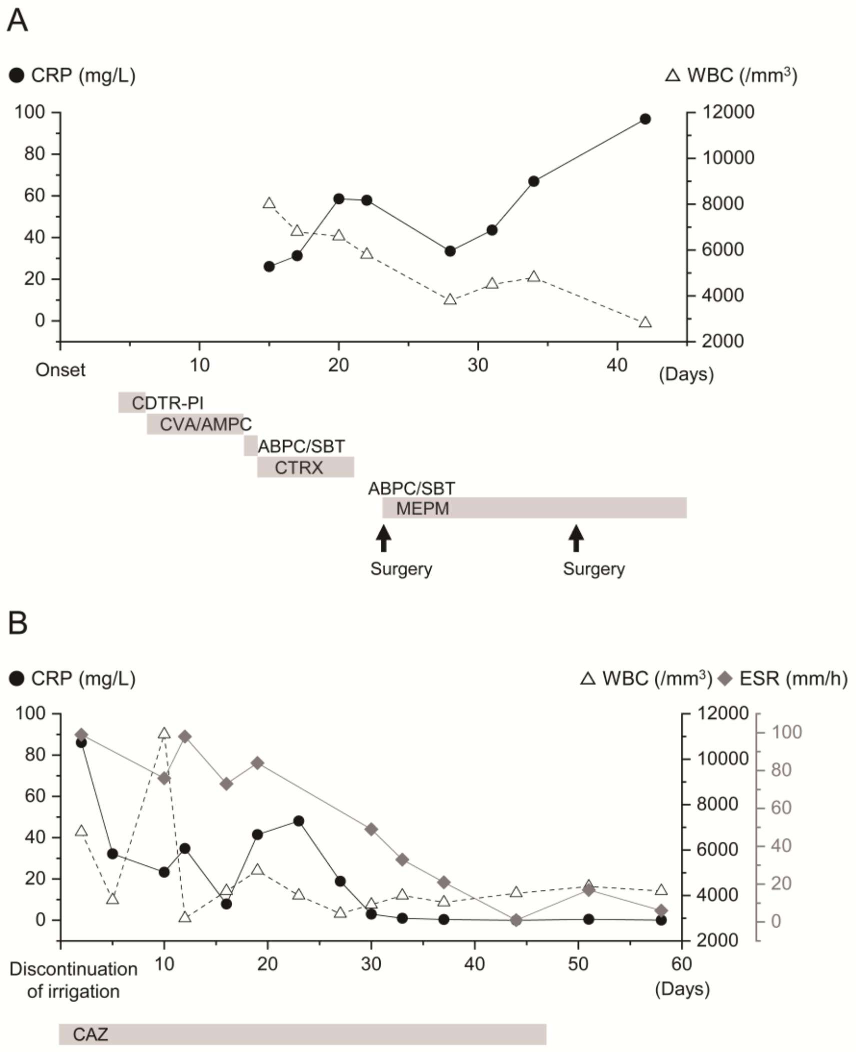

Supplement: Supplementary file 1 [file medi-102-e33012-s001.pdf]
